# Interspecific Germline Transmission of Cultured Primordial Germ Cells

**DOI:** 10.1371/journal.pone.0035664

**Published:** 2012-05-21

**Authors:** Marie-Cecile van de Lavoir, Ellen J. Collarini, Philip A. Leighton, Jeffrey Fesler, Daniel R. Lu, William D. Harriman, T. S. Thiyagasundaram, Robert J. Etches

**Affiliations:** 1 Crystal Bioscience Inc, Emeryville, California, United States of America; 2 Department of the President's Affairs, Management of Nature Conservation, Abu Dhabi, United Arab Emirates; University of Bonn, Institut of experimental hematology and transfusion medicine, Germany

## Abstract

In birds, the primordial germ cell (PGC) lineage separates from the soma within 24 h following fertilization. Here we show that the endogenous population of about 200 PGCs from a single chicken embryo can be expanded one million fold in culture. When cultured PGCs are injected into a xenogeneic embryo at an equivalent stage of development, they colonize the testis. At sexual maturity, these donor PGCs undergo spermatogenesis in the xenogeneic host and become functional sperm. Insemination of semen from the xenogeneic host into females from the donor species produces normal offspring from the donor species. In our model system, the donor species is chicken (Gallus domesticus) and the recipient species is guinea fowl (Numida meleagris), a member of a different avian family, suggesting that the mechanisms controlling proliferation of the germline are highly conserved within birds. From a pragmatic perspective, these data are the basis of a novel strategy to produce endangered species of birds using domesticated hosts that are both tractable and fecund.

## Introduction

Of the 5000 animal species protected by the Convention on International Trade in Endangered Species (CITES), approximately 28% are birds (www.cites.org). One hundred and fifty two species are threatened by extinction and another 1268 species are protected by regulating international trade. The global emphasis in conservation programs is on protection of environments, restriction of trade and hunting, and propagation of captive populations with or without a release component. In spite of this intense global effort, populations of many threatened species continue to decline. The global effort to propagate endangered birds would be enhanced if the reproductive capacity of these species could be augmented using surrogate parents from domestic birds that are much more tractable and fecund. Execution of this concept requires two components. Firstly, a cell culture system capable of expanding the small population of PGCs in rare embryos of a threatened species is obligatory. Secondly, the germline of the donor species must be transmitted through the germline of the host species.

The phenomenon of colonization of the xenogenic gonad was first demonstrated using turkeys and chickens [1] and corroborated in toads [2], birds [3]–[7] and fish [8]. In toads and fish, robust levels of germline transmission of xenogeneic PGCs are apparent [2], [9], [10] but in birds, the frequencies of chimera production and germline transmission are low [11]–[14] and frequently rely on a single embryo as evidence of germline transmission. Only 2 males, out of 65 evaluated, sired more than 1 offspring [11]–[13]. All reports on interspecies germline transmission describe transmission of male chicken PGCs through the male germline of a xenogenic species. The breeding to determine this germline transmission uses sperm collected from the xenogeneic species and inseminated into hens. As a result, the chicken offspring hatches out of a chicken egg. Unambiguous evidence of xenogeneic germline transmission, therefore, requires rates of germline transmission that are well above the 6–15% error rate of pedigree analysis, see table 1 in [15] and/or an internal marker that verifies transmission of the donor genome. To evaluate colonization and transmission rates, we use a culture of GFP expressing primordial germ cells allowing us to track the donor cells through the germline of the xenogeneic host.

**Table 1:** Evaluation of sperm by flow cytometry and production of GFP-expressing and naked neck offspring from interspecific male guinea fowl chimeras.

male ID	cell line	GFP sperm %	fertility	GFP transmission (%)	naked neck transmission(%)	hybrids (of total hatched)
311	167.2	0	-	-	-	-
314		26	18%	11/35 (32)	16/28 (57)	5/33
790		0	-	-	-	-
270	169.4	0	-	-	-	-
271		20	-	-	-	-
279		0	-	-	-	-
289		0	-	-	-	-
291		0	-	-	-	-
308		56	74%	42/95 (44)	24/60 (40)	1/61
317		78	35%	23/45 (51)	20/37 (54)	0/37
323		7	-	-	-	-
334		0	-	-	-	-
524		71	15%	8/20 (40)	9/20 (45)	0/20
802		78	38%	26/55 (47)	19/41 (46)	0/41

The GFP and naked neck loci are heterozygous and therefore, one half of the offspring from the injected PGCs inherit the expressed alleles at these loci. The high rates of fertility indicate that the testis is highly colonized by chicken PGCs in some of the interspecific male guinea fowls. Hybrids appear in the offspring of roosters with lower rates of germline transmission. GFP transmission is calculated from all embryos evaluated including embryos at D7 and chicks at hatch. Naked neck transmission was evaluated in hatched chicks and the hybrid percentage was calculated as a percentage of all offspring including both chicks and hybrids.

## Results

**Figure 1:**
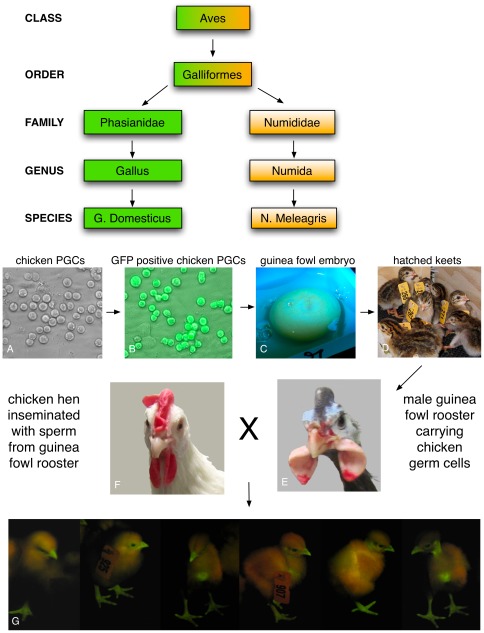
Xenogeneic offspring from guinea fowl. Upper panel: Phylogenetic relationship between chicken (Gallus domesticus) and guinea fowl (Numida meleagris). Bottom panel: primordial germ cells migrating through the blood in a 2.5 day old embryo were retrieved and cultured (A) and transfected with a GFP expression construct. GFP positive PGCs (B) were injected into guinea fowl embryos (C). The embryos were hatched (D) and males were raised to sexual maturity (E). The guinea fowl males were mated to White Leghorn females (F) and GFP positive chicken offspring (G) were obtained demonstrating that the chicken PGCs had passed through the germline of the male guinea fowl.

The provenance of xenogenic offspring from guinea fowl males was determined using the protocol illustrated in Figure 1.

**Figure 2:**
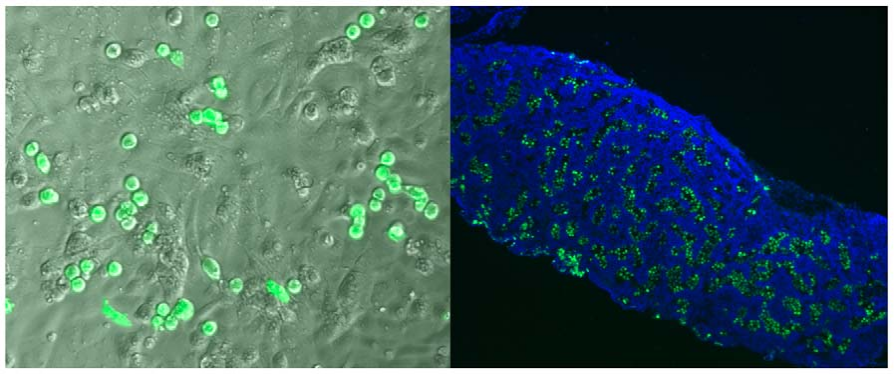
Colonization of xenogeneic testis (Left panel) GFP positive chicken primordial germ cells in culture on irradiated BRL feeder cells. (Right panel) To evaluate the incorporation of chicken GFP positive cells into the gonads of guinea fowl, testes were retrieved, fixed in 4% paraformaldehyde and processed for cryosectioning. GFP expressing cells are present only in the seminiferous tubules suggesting that they have differentiated into spermatogonia. The absence of GFP-expressing cells in the interstitial tissue is consistent with the exclusive commitment of PGCs to the germline. Sizebar denotes 100 µm

Two male chicken (Gallus domesticus) PGC cell lines were derived from single embryos heterozygous for the dominant naked neck gene (Nana) [16]. The cell lines were transfected [17], and clones expressing green fluorescent protein (GFP) were selected (Fig. 2A). Two clones were expanded, one from each parental cell line (169.4; 143–151 days in culture and 167.2; 138 days in culture) and used to inject 141 guinea fowl (Numida meleagris) embryos at the equivalent of Stage 13–15 [18].

Following injection of the in-vitro produced chicken PGCs into guinea fowl embryos after 60 h of incubation, colonization of the guinea fowl gonad by the GFP-expressing chicken PGCs was examined histologically in embryos sacrificed at various times during incubation and in the peri-hatching period. All 12 embryos examined revealed that GFP-expressing PGCs were incorporated into the gonad (Figure 2B).

A total of 51 embryos hatched and a cohort of 14 male putative interspecific germline guinea fowl chimeras was reared to sexual maturity. The 37 embryos not retained for germline testing comprised females, males whose testis were examined histologically for evidence of incorporation of GFP-expressing germ cells and keets that died before sexual maturity. The sex ratio was unaffected by the introduction of male germ cells.

Semen was collected from the 14 chimeras reared to sexual maturity and analyzed for GFP expression by flow cytometry (Table 1). Semen from 5 of the putative interspecific germline guinea fowl males contained between 26% and 78% GFP-expressing sperm suggesting that the in vitro produced chicken PGCs had extensively colonized seminiferous tubules in the guinea fowl testis and were actively undergoing spermatogenesis. These five males were mated to female chickens. Their eggs were collected, incubated and either hatched or opened during development (E7) to evaluate the presence or absence of the naked neck phenotype and GFP expression (Table 1). Because both the naked neck and GFP loci were heterozygous for the expressed alleles, the rate of germline transmission of the injected chicken PGCs is twice the rate of GFP or naked neck transmission. As shown in Table 1, approximately 50% of the offspring express either GFP or naked neck, indicating that most of the offspring were the product of the union of chicken eggs with a chicken sperm.

**Figure 3:**
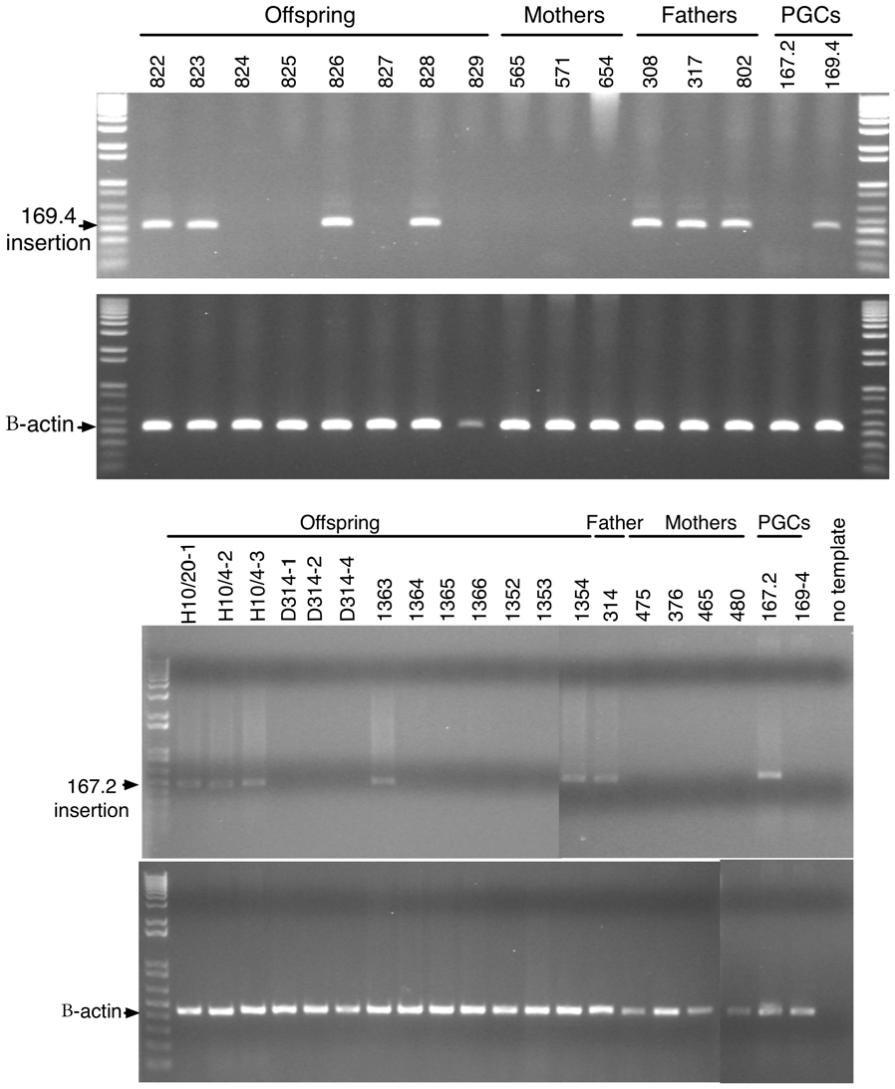
Pedigree analysis of offspring. PCR amplification of genomic DNA from combs of chicks, blood from their wild-type mothers, semen from their interspecific chimeric fathers and the 169.4 PGC and 167.2 cell lines that were incorporated into the testis of the interspecific chimeric males. The GFP locus in the 169.4 and 167.2 is heterozygous and therefore, approximately one half of the offspring from the injected PGCs inherit the locus and express GFP. Top panel, offspring from the 169.4 cell line. Bottom panel; offspring from the 167.2 cell line.

The provenance of the GFP offspring from the in vitro-produced chicken PGCs was confirmed by sequencing the site of insertion of the GFP locus in both the cell line and the GFP-expressing chicks sired by the interspecific chimeric males. Integration site-specific PCR assays were developed for typing the offspring. In each case, the insertion site of the GFP locus in the genome of the offspring was identical to that of the PGC line inserted into the germline of the interspecific sire, confirming the functionality of sperm produced in a xenogenic testis and derived from in vitro cultured PGCs. Examples of this analysis are shown in Figure 3.

Interspecific hybridization of birds is frequent and well documented [19]. Accordingly, the production of hybrids was estimated from each of the five matings of chimeras to chickens by the absence of a comb and hatching after 22 days of incubation as opposed to 21 days for chickens. Of the 192 offspring hatched from the interspecific chimeric guinea fowl males, only 6 were interspecific hybrids.

The strategy of using interspecific chimeras to propagate endangered species of birds requires that the offspring are reproductively normal. Accordingly, offspring from the interspecific chimeras were reared to sexual maturity, mated to wild-type chickens and their reproductive performance was recorded. The average fertility and hatching rate of the 5 hens tested was 71% and 94%, respectively and of the 4 roosters tested was 78% and 87% respectively, indicating that the offspring is reproductively normal.

## Discussion

Using chickens as a donor species and guinea fowl as hosts, we have demonstrated feasibility of the essential components of a strategy to propagate endangered species of birds. We show that PGCs from a single donor chicken embryo can be expanded a million fold in vitro. Following transfer into domesticated guinea fowl, a species that belongs to the same order but to a different family (Figure 1), these PGCs develop into functional sperm. We have used GFP expression as an internal marker to verify colonization of the in vitro produced PGCs in the developing testis and as the testis developed, histological analysis showed that the PGCs were located in their appropriate physiological compartment. Finally, by pedigree analysis we showed that the rates of germline transmission are robust and two to three times greater that the highest error rates from pedigree analysis. Taken together, these data are compelling evidence to support a strategy for propagating endangered species of birds using surrogate inter-specific germline chimeras even when the species are from different phylogenetic families.

In previous studies [11]–[14], both the frequency of germline chimeras and of germline transmission, were low. By contrast, 50% of our roosters were chimeric as determined by the presence of GFP positive sperm in the semen. Fertility in our study ranged from 15% to 74% with very few hybrids produced. These data demonstrate that the germline transmission rate of interspecific chimeras can be raised above levels that are phenomenological to levels that are practical. Furthermore, the 192 offspring were produced during a three week period. Extrapolation of these numbers to endangered species suggests that thousands of birds could be produced from a small flock of approximately 100 interspecific chimeric domesticated birds.

In birds, interspecific hybrids are observed frequently. Hence, our examination of the feasibility of using interspecific chimeras to propagate endangered species of birds incorporated GFP expressing PGCs to unequivocally track the donor genome. The presence of GFP in offspring of the mating between interspecific chimeric guinea fowl males and chickens is a surrogate for the entire *Gallus gallus* genome. In the absence of a GFP marker, neither the provenance of the offspring nor their pedigree could be determined unambiguously. Conversely, the presence of GFP indicates that the entire haploid *Gallus gallus* genome was transmitted through the *Numida meleagris* host. The veracity of this data was corroborated by confirming that the insertion site of the GFP transgene in offspring was identical to the insertion site in the PGCs that were introduced into the interspecific chimeric guinea fowl at day 3 of development.

Previous attempts to validate the idea of using interspecific chimeras to propagate endangered species of birds have relied on the transfer of freshly collected PGCs. However, this approach is impractical because many donors are required to collect a sufficient number of PGCs for introduction into a single host, a significant number of injected hosts fail to develop and most of the putative interspecific chimeras do not transmit through the germline. For example, Wernery et al [13] used an unknown number of embryos from an endangered bustard to create a single offspring. In the study of Kang et al [11], at least 90 embryos were sacrificed in order to make two germline transmitting males. Without a cell culture system that provides an essentially unlimited supply of PGCs, the approach is not feasible because more embryos are sacrificed than produced.

Although this test case using two abundant species of domestic birds demonstrates that interspecific chimeras could be used to propagate endangered species of birds, widespread adoption of the strategy requires additional components. Firstly, pan-avian culture conditions to derive large populations of PGCs from individual embryos of endangered species is needed. In addition, both male and female populations of PGCs need to be established since germline transmission of PGCs requires that the sex of the donor be matched to the sex of the recipient [17], [20]. Additional data is also required to evaluate the effect of xenogeneic yolk, albumen and shell on embryonic development. Like birds, amphibian embryos utilize maternal yolk and albumen to support embryonic development. In interspecific chimeras formed by introducing *Xenopus mulleri* PGCs into *Xenopus laevis* hosts, development of *X. mulleri* embryos was not impaired [2] suggesting that yolk, albumen and shell, like milk, can be substituted across species. A recent anecdotal report of a houbara bustard chick hatching from a chicken egg is consistent with this prediction [21] and suggests that the physiological properties of eggs are highly conserved among birds.

## Materials and Methods

### PGC derivation and culture

PGC cell lines were derived and expanded according to [17]. Blood was retrieved from Stage 14–16 H&H [18] embryos and put individually into culture on a feeder layer of irradiated Buffalo Rat Liver (BRL) cells. The medium consisted of Knock-Out DMEM (Invitrogen), of which 40% was preconditioned on BRL cells [22] supplemented with 7.5% FBS (standard FBS, Hyclone), 2.5% irradiated chicken serum, 2 mM glutamax, 1× non-essential amino acids, 1 mM pyruvate, 0.1 mM β-mercapto-ethanol (all Invitrogen), 6 ng/ml rmSCF and 4 ng/ml rhFGF (R&D Systems, Minneapolis, MN). The medium was changed every few days and the cells were passaged to new wells every 4–7 days. A population of PGCs becomes visible after 7–10 days and is expanded under the same culture conditions. Two parental cell lines were used for the transfections.

### eGFP construct and transfection

Two eGFP containing constructs were used to create GFP-expressing cell lines. Both constructs consisted of a chicken β-actin promoter driving expression of the enhanced green fluorescent protein gene and the CAG promoter (the chicken β-actin promoter and a CMV IE enhancer, gift from Connie Cepko, Harvard Medical School) driving expression of resistance to puromycin. An attB site was included to allow site-specific recombination with a pseudo-attP site in the genome facilitated by integrase [23]. One of the constructs also contained HS4 insulator elements around the selectable marker [23]. For each transfection 5×10^6^ cells were collected, pelleted and resuspended in V-buffer (Lonza Walkerville, Walkerville, MD) with 15–20 µg of circular construct and 7.5–10 µg of CMV-integrase (kindly provided by Michelle Calos, Stanford University) for a total volume of 100 µl. The cell-DNA suspension was transferred to a 1 mm cuvette and subjected to 8 square wave pulses of 350 V/125 µsec (BTX 830 electroporator). The transfected cells were resuspended in medium and plated into a full 48 well plate with BRL feeder cells. Selection with 1 µg/ml of puromycin was started 4–5 days following transfection. Throughout selection, medium was changed regularly. As soon as GFP expressing colonies were identified they were transferred to new wells and expanded for injection.

### Production of interspecies chimeras

3000 GFP-expressing chicken PGCs were injected into the vasculature of guinea fowl embryos at the equivalent of stage 13–15 H&H. After injection the embryo was transferred to a surrogate shell and incubated for 25 days until hatch.

### Flow cytometry

Sperm was collected from the chimeric guinea fowl roosters and diluted with PBS/0.5% FBS to obtain a homogenous suspension. The suspensions were filtered through the strainer caps of BD Falcon 5 ml polystyrene tubes. GFP-positive and -negative sperm were used as controls.

### Pedigree analysis

Genomic DNA from the injected PGC lines was digested with an enzyme that left the eGFP constructs intact, re-ligated, transformed into E. coli, amplified under ampicillin selection and the sequence of the flanking genomic DNA adjacent to the eGFP construct was determined. The sequence was used to evaluate the site of integration into the chicken genome and to derive site-specific PCR primers [23]. Both the 169.4 insertion and the 167.2 insertion were in repetitive elements and the chromosome location could not be determined.
